# Determining the Oxidation Mechanism through Radical Intermediates in Polysorbates 80 and 20 by Electron Paramagnetic Resonance Spectroscopy

**DOI:** 10.3390/ph17020233

**Published:** 2024-02-09

**Authors:** Adam T. Sutton, Richard R. Rustandi

**Affiliations:** Analytical Research & Development, Merck & Co., Inc., Rahway, NJ 07065, USA; richard_rustandi@merck.com

**Keywords:** polysorbates, oxidation, radical intermediates, reaction mechanism, electron paramagnetic resonance spectroscopy

## Abstract

Polysorbates 20 and 80 (PS20 and PS80) are added to many commercial biologic and vaccine pharmaceuticals. It is commonly known that these polysorbates undergo a radical oxidation mechanism; however, the identity of these radical intermediates has not been clearly determined. Furthermore, PS20 and PS80 differ by the presence of a lauric acid instead of an oleic acid, respectively. The oxidation of PS80 is thought to be centered around the double bond of the oleic acid even though PS20 also undergoes oxidation, making the mechanism of oxidation unclear for PS20. Using commercial stocks of PS20 and PS80 alkyl (R^•^), alkoxyl (C-O^•^) and peroxyl (C-OO^•^) radicals were detected by electron paramagnetic resonance spectroscopy likely originating from radical-initiating species already present in the material. When dissolved in water, the peroxyl radicals (C-OO^•^) originally in the stocks were not detected but poly(ethylene oxide) radicals were. An oxidative pathway for polysorbates was suggested based on the radical species identified in the polysorbate stock material and solutions.

## 1. Introduction

**Figure 1:**
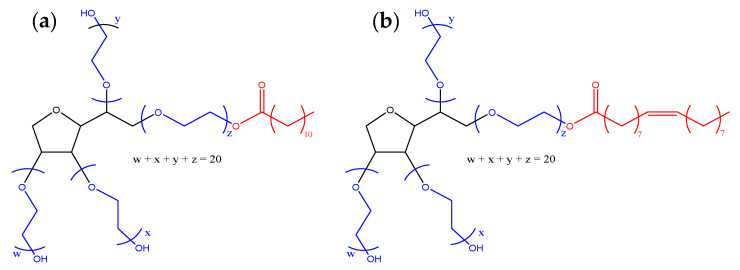
Target chemical structure of (**a**) polysorbate 20 and (**b**) polysorbate 80. The polyethylene oxide (PEO) chains are shown in blue; the fatty acid is shown in red, and the sorbitol group is shown in black.

Polysorbates (PSs) are used in various pharmaceuticals [1], vaccines [2] and food [3] products as stabilizing agents [4]. Commercial PSs are produced from the esterification of fatty acids with sorbitol which is further reacted with ethylene oxide [5], producing a mixture of molecules varying in the type of fatty acid attached, the number of fatty acids attached and the lengths of the poly(ethylene oxide) (PEO) chains, also referred to as poly(ethylene glycol) (PEG) chains (Figure 1) [6].

Being such a complex mixture of molecules, it is possible to observe batch-to-batch variability in the PS product both in the certificate of analysis and the performance or lifetime of the PSs. The variability in PS products is important to control since the PS is known to degrade due to both hydrolysis and oxidation, as demonstrated several times in the literature [7,8,9,10,11]. The degradation has been reported to be influenced by several factors including the storage solution, exposure to heat or light, presence of metals, exposure to oxygen and the chemical makeup of the PS itself [12,13,14,15]. Due to the plethora of factors influencing the potential degradation of PSs and thus affecting the commercial products in which they are used, there has been great interest from academia and industry to better understand the mechanism of degradation of PSs to better prevent the degradation from occurring. Moreover, the two most commonly used PSs are polysorbate 20 and polysorbate 80, referred to as PS20 and PS80. These polysorbates differ in their fatty acid group, where PS20 contains predominantly the saturated lauric acid and PS80 mainly contains a monounsaturated oleic acid. The oxidation mechanism for PS80 has often focused on the presence of the double bond in the unsaturated fatty acid [16]; however, similar oxidation products can be found in PS20, suggesting that their oxidation mechanisms might involve similar pathways [17]. Furthermore, a recent industry perspective has mentioned the lack of understanding in the oxidation of PS20 [18]. Therefore, additional information regarding the differences and similarities in the oxidation mechanism of PS20 and PS80 is needed.

While the hydrolysis degradation mechanism of PS is not complex, the oxidative degradation mechanism has been proposed in multiple variations [7,8,10,11,17,19,20]. All of the proposed mechanisms for oxidative degradation involve a radical mechanism even though the detection of these radical intermediates has either been unsuccessfully attempted [12] or there has been a lack of detection of the individual radical species [21]. Typically, mass spectrometry (MS) [8,11,22] or modeling predictions [20] have been used to identify non-radical intermediates and final oxidative products to propose a potential mechanism. Since all mechanisms suggest that radical intermediates take part in the oxidation, techniques that specifically study organic radicals are needed to identify the radical intermediates.

The detection of radical intermediates is very challenging as radical species typically have a half-life in the range of microseconds [23]. The short half-life also means that measurements need to be taken as the radicals are forming. To extend the half-life of the radical species, making them more easily observed by analytical techniques, spin traps are often added. Spin traps react readily with radical species to produce a more stable radical species which usually have a half-life in the range of seconds to hours [23]. Several spin traps exist; some common examples include alpha-phenyl N-tertiary-butyl nitrone (PBN), 5-Diisopropoxyphosphoryl-5-methyl-1-pyrroline-N-oxide (DIPPMPO) and 5,5-dimethyl-pyrroline N-oxide (DMPO). DMPO is the most commonly used spin trap as it has the largest database of known DMPO radical adducts [24]. To identify a radical adduct and thus the radical intermediate structure, characterization techniques such as nuclear magnetic resonance (NMR) spectroscopy [25] or MS [26] can be used; however, these are only applicable to very stable adducts that can last the duration of the measurement. Electron paramagnetic resonance (EPR) spectroscopy, also known as electron spin resonance (ESR) spectroscopy, is most commonly used to identify radical species as it can measure all radical species simultaneously while also differentiating each radical based on the splitting pattern and hyperfine coupling constants (hfc) of the signals. The splitting pattern and hfc can then be compared to known databases to confirm the identity of the radical formed.

Herein, EPR spectroscopy was used to identify the types of radical intermediates present in the PS products, PS20 and PS80. Pure stocks of PS20 and PS80 were examined to identify the radical intermediates that can form in PS raw materials which are indicative of the oxidation that would occur in any stored PS material. PSs are typically used as an aqueous solution and most reported degradation mechanisms are of PSs in aqueous solutions; therefore, 10% w/w aqueous solutions made from the same pure stocks were also analyzed to compare the different radical species present when in water and when stored as a pure stock. The identity of the radical species was confirmed by comparing the obtained hfc to the published hfc for radical adducts that had previously been identified in similar chemical structures. Ultimately, an oxidative degradation mechanism is suggested to explain what is observed in the use of raw PS materials and solutions when they are subsequently used in pharmaceutical products as aqueous solutions based on the radical species identified in the PS samples.

## 2. Results

### 2.1. EPR Spectroscopy Method Optimization

**Figure 2:**
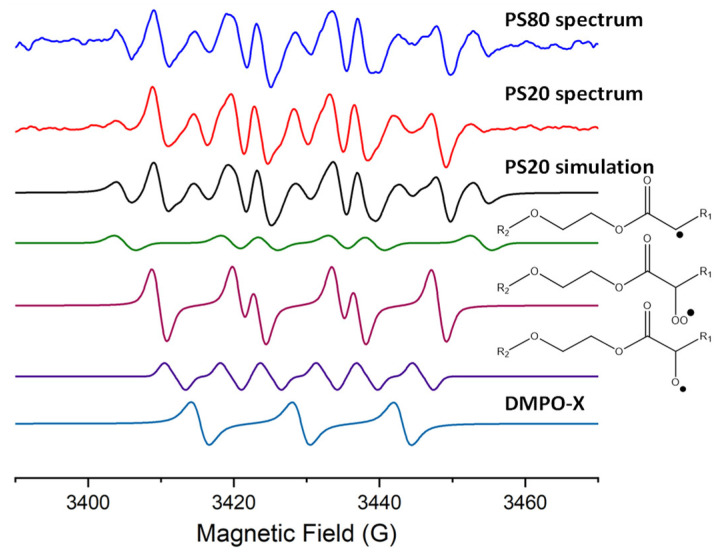
EPR spectra of neat PS20 and PS80 stocks with DMPO, as well as simulations of spectra from DMPO radical adducts of R^•^, R-OO^•^, R-O^•^ and DMPO-X.

Stock liquids of PS20 and PS80 were mixed with the spin trap 5,5-dimethyl-1-pyrroline-*N*-oxide (DMPO) to capture and help identify the radicals present. Measuring the stock solutions at room temperature (20–23 °C) or in the presence of UV light resulted in no radicals being detected. However, when the temperature was increased to 50 °C, radicals were detected (Figure 2) with EPR and the signal was optimized in terms of EPR parameters such as modulation amplitude, the number of scans and gain with a measurement time of 500 s. In order to fit the observed PS spectra, the splitting patterns of the same four radical species were required for both PS20 and PS80 liquid stocks. This indicates that there are radical-producing species present in the commercial stocks and that the radical mechanism was initiated by the elevated temperatures. Oxidative mechanisms for PSs usually start with a PS molecule losing a hydrogen due to UV, temperature or metals producing an alkyl radical. It is rarely acknowledged in the proposed mechanisms that the commercial PS product already contains peroxides and, as such, oxidation can occur without first generating an alkyl radical [11,16,17]. Peroxide species have been observed in commercial PS stocks [27], as well as their components, PEO [28] and free fatty acids. Additionally, hydrogen peroxide can be added in the production of polysorbates and remain in the commercial product [5]. Moreover, in the modeling of the kinetics of oxidation of PS20 and PS80, it was found that the decomposition of peroxides was the only appropriate way to fit the radical initiation step, supporting that oxidation is initiated by the peroxide species already present in the material [29]. Therefore, the presence of radical-initiating species in the commercial stocks was expected.

The concentration of each radical species can be determined from the peak area of the simulated fitting of each radical in the EPR spectrum. The calculation can be directly performed by SpinCount in the Xenon software. However, there are several optimizations that must be made in the measurement in order to obtain meaningful concentrations. Some of the optimizations needed are discussed by Mittag et al. [21]. Firstly, PS stocks are very viscous, so an excess of spin trap is needed as well as being thoroughly mixed throughout the sample to ensure all radical species are captured. The sensitivity of the signals must also be sufficient to reduce the variability in the fitting of the spectra. Without optimizing the sensitivity of our EPR spectroscopy measurement, the signal-to-noise ratio (SNR) was such that there was approximately 10–20% variation in the peak area when trying to refit the same spectrum. From the amount of sample measured in this study, with 25 scans, the limit of quantification (as in when a consistent peak area could be fitted without peak area variation) was approximately 1 µM for an individual radical species. The main challenge observed with obtaining accurate quantification of radicals was that the samples needed to be heated to 50 °C to detect the radical with sufficient SNR; however, the duration of heating impacted the absolute quantity of radicals. Additionally, when monitoring PS samples with DMPO present overtime at 50 °C, it was observed that the concentrations of each radical species did change overtime. The change occurred over hours; therefore, to provide meaningful ratios of each species, careful planning is needed to ensure that the samples introduced to the EPR instrument are exposed to the same temperature for the same duration of time. Considering the temperature stability of the heating apparatus used, there would be a degree of variability in the quantitative measurements obtained, and thus, the ratios of the radical species determined should be considered semi-quantitative.

### 2.2. Radical Adduct Identification

Radical adducts of DMPO have characteristic splitting dictated by hyperfine coupling constants (hfc) which allow for the identification of specific radical species. DMPO was the spin trap used for this study as it was the only spin trap with previously identified adducts associated with PEO and fatty acids which are present in PS. Analysis of the PS stocks and solutions without DMPO resulted in no detectable signals. The identification of each radical species is summarized in Table 1. Peroxyl (R-OO^•^) and alkoxyl (R-O^•^) radicals were detected, which are common radicals observed in fatty acids (Figure 2) [30,31]. The difference in the hfc of a peroxyl or alkoxyl radical attached to the fatty acid or PEO parts of the PS molecule would be too small to distinguish [32]. However, previous attempts to observe alkoxyl radicals in pure PEO by EPR have failed as these radicals are said to degrade faster than their reaction with DMPO [28]. Additionally, alkyl radicals on PEO were calculated to react magnitudes faster with DMPO than with oxygen, preventing the formation of peroxyl radicals, meaning only peroxyl radicals on PEO prior to initiation could be detected [28]. Since no radicals were detected in the polysorbates unless they were heated to 50 °C, it is likely that the majority of the peroxyl or alkoxyl radicals detected are associated with those on the fatty acids of the PS and not the PEO chains, although alkoxyl radicals on the PEO chains could be present and not detected with the DMPO spin trap. An alkyl (R^•^) radical was identified in the EPR spectra of the stock PS. Alkyl radicals on the fatty acid group and the PEO chains differ by the presence of an oxygen adjacent to the carbon-centered radical. This distinction creates differences in the hfc reported for alkyl radicals in fatty acids and PEO. DMPO alkyl radical adducts in fatty acids have been reported to have hfc of *a*_N_ = 14.34 G, *a*_H_ = 20.89 G (in pure oils at various temperatures) [33], while for PEO, they have been reported as *a*_N_ = 15.8 G, *a*_H_ = 21.8 G (in water and at various temperatures) [28]. This slight but noticeable difference indicates that the predominant alkyl radical is on the fatty acid group as the hfc determined was *a*_N_ = 14.7 ± 0.2 G, *a*_H_ = 19.8 ± 0.3 G (n = 10). Any alkyl radicals on the PEO chains would be in a smaller quantity below the limit of detection. A three-line signal was also detected which has been identified when DMPO has been used in oxidative environments, generally referred to as DMPO-X, and has been observed in EPR spectra of PEO before (Figure 2) [28]. DMPO-X is mostly likely the disproportionation or oxidation of a DMPO adduct; due to a three-line signal being present, the suggested chemical structure for DMPO-X is the replacement of the alpha hydrogen with an alcohol group [34]. The presence of DMPO-X also impacts the accuracy of comparing the ratios of different radical species as it is unknown if certain adducts are more susceptible to oxidation to form DMPO-X.

Interestingly, the same radicals were identified for PS20 and PS80 in similar proportions (Figure 2 and Figure S1). PS20 and PS80 differ in their fatty acid composition. PS20 contains primarily the 12-carbon saturated fatty acid, lauric acid, in contrast to PS80 which mainly contains the monounsaturated oleic acid. The presence of the carbon–carbon double bond has led to most studies on the oxidative mechanism of PS being focused on PS80. Some studies have demonstrated that unsaturated fatty acids both free and in PS molecules will have an increased rate of oxidation compared to their saturated counterparts [20,33]; nevertheless, saturated fatty acids also undergo oxidation [30]. In the case of PS20 and PS80, the final oxidation products observed are usually similar, indicating that the carbon–carbon double bond may change the oxidative intermediates (such as radical addition to the double bond producing the alkyl radicals observed), but the final product (cleaved PEO chains and fatty acids) will be the same. The EPR spectra of the PS20 and PS80 stocks contained the same radicals, indicating that if radicals adjacent to the carbon–carbon double bond were present, then they were below the limit of detection.

**Figure 3:**
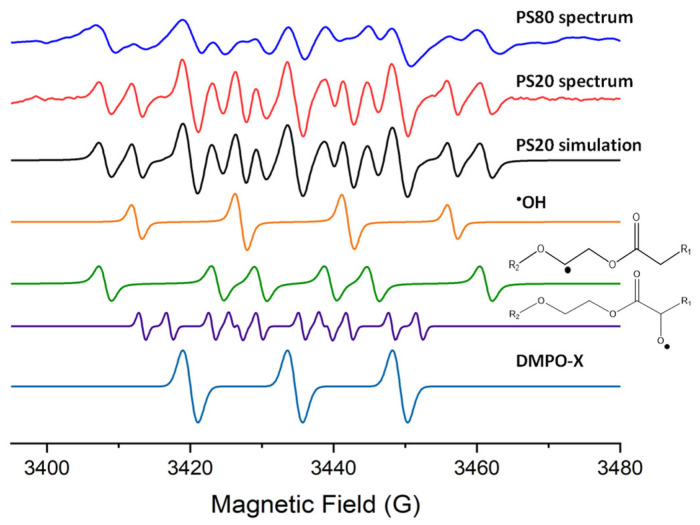
EPR spectra of 10% aqueous PS20 and PS80 with DMPO, as well as simulations of spectra from DMPO radical adducts of ^•^OH, R^•^, R-O^•^ and DMPO-X.

When 10% w/w aqueous solutions of PS20 and PS80 were measured by EPR spectroscopy at 50 °C, no peroxyl radicals were identified (Figure 3 and Figure S1). The alkoxyl (R-O^•^) radicals in the stocks were typically 15–25% of the radical concentration in the spectra. The same alkoxyl (R-O^•^) radicals were identified in the 10% w/w aqueous solutions but typically at 2–10% of the radical concentrations (Figure 3). The hfc of the fitted alkoxyl (R-O^•^) radical between the stocks and the aqueous solutions do differ by 5–10%. Different alkoxyl radicals are reported to have greater variation in the hfc than alkyl radicals, so this change in hfc might indicate a change in the predominate alkoxyl radical location [32,35]. Although the reason could be due to the presence of water, which would impact alkoxyl radicals more than alkyl radicals due to hydrogen bonding, nevertheless, the identified hfc are within the range of hfc reported previously for alkoxyl radicals [30,32,35]. It was typically observed in the solutions that more than 15% of the radical concentration was hydroxyl (^•^OH) radicals (Figure 3). The other radical identified in the solutions was an alkyl (R^•^) radical; however, the hfc had changed compared to the alkyl (R^•^) radical observed in the stock mixtures (Figure 2). The hfc were *a*_N_ = 15.8 ± 0.1 G, *a*_H_ = 22.3 ± 0.3 G (n = 6), which are very similar to the reported hfc of PEO alkyl radicals, suggesting that in aqueous solutions, the predominant alkyl radical is those on the PEO instead of on the fatty acid group, as in the commercial stocks.

## 3. Discussion

**Figure 4:**
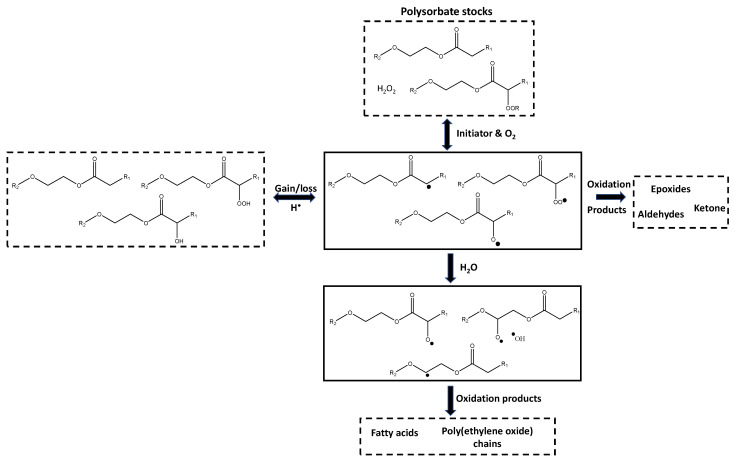
Proposed oxidation pathway for PSs. Full-line boxes indicate intermediate radical species were detected. R_1_ is the saturated and unsaturated alkyl chains of the fatty acids in the PS. R_2_ is the sorbitol and PEO groups of the PS (see Figure 1). The black dots show the position of the radical.

From the identification of these radical species, the major oxidation pathway in PSs could be constructed (Figure 4). It is important to note that other oxidation pathways may occur simultaneously, but from the radical intermediates observed, the most common pathway is proposed. The initial step in oxidation typically starts with a PS molecule, but since there are already peroxide species in the commercial PS stocks, the initial step would involve the activation of these peroxide species rather than the abstraction of a hydrogen from the PS molecule. That is because at 20–23 °C, no radical species could be detected; however, when the temperature increased to 50 °C, multiple radical species were identified. The enthalpy of dissociation of hydroperoxides in PEO was reported to be 148.5 kJ/mol at 50 °C [38]. The addition of oxygen to PEO has been observed at 50 °C; however, it was only detectable after 30 min of oxygen exposure [39]. For the oxidation of fatty acids, significant increases in radical species were not detected until 130 °C was reached, suggesting the dissociation of alkyl chain or transfer to oxygen due to the increased temperature is not prevalent until temperatures much higher than 50 °C are reached [35]. At elevated temperatures such as 50 °C, the peroxides are initiated, and the radical species are propagated. This is in agreement with the reported self-accelerating decomposition temperatures for organic peroxide which are typically in the range of 25–50 °C; for lauroyl peroxide, which would be one of the mostly likely peroxides in PS20, the self-accelerating decomposition temperature was reported as 46 °C [40]. The carbon–carbon double bond in PS80 may increase the rate of oxidation, as observed in previous studies of polysorbates [16,41] and fatty acids [42,43]; however, it does not seem to be related to the initiation of the radical species based on the radicals observed in this study.

Based on the EPR spectra of the stocks, the radicals propagating consist mainly of alkyl (R^•^), peroxyl (R-OO^•^) and alkoxyl (R-O^•^), which is consistent with the radicals reported for PSs [21], most likely on the fatty acid part of the PS molecules. No ^•^OH radicals were detected, which could be due to no hydrogen peroxide being present in the batches analyzed or because the hydrogen peroxide quickly reacts to form the other radicals. The alkyl radical could form from the abstraction of a hydrogen from the other propagating radicals [7,11]. As oxygen enters the system, it can react with the alkyl or alkoxyl radicals to generate additional peroxyl radicals or it may be produced from the loss of a hydrogen from hydroperoxide. The alkoxyl radical is likely produced from the dissociation of the peroxides. Nevertheless, these radicals would be propagating and converting between each other until termination reactions are achieved. Various degradation products have been identified in PSs which are the results of several possible termination reactions. For PS80, aldehyde, ketone and epoxide species are regularly detected [10,44].

When the PS is in an aqueous environment, transfer of the radical to water occurs easily, as shown by the formation of hydroxide radicals, or to the buffer components such as histidine, as recently demonstrated by the detection of histidine radicals using EPR spectroscopy in Zheng et al. [27]. The peroxide and alkyl radicals of the fatty acid part of the PS were no longer present in detectable quantities, mostly likely because they easily transfer to the water molecules or due to the decomposition of the peroxyl radicals. PEO molecules have been used previously to protect molecules from hydroxyl radical attack and in the process, produce alkyl radicals on the PEO chains [28,36,37]. Therefore, this transfer of hydroxyl radicals to PEO radicals is likely occurring in PS solutions as well. The PEO radicals are the sources of significant degradation to PS molecules as they can undergo beta-scission, breaking apart the molecule releasing PEO chains, free fatty acids [10,11,15,43]. Additional hydroxyl radicals in the storage solutions of PS can be produced from various sources, as identified in literature, such as oxygen, UV light and metals [12,13].

## 4. Materials and Methods

Polysorbate 20, labeled as Tween 20, was purchased from Sigma (Allentown, PA, USA). It contained 53% lauric acid, with the other major fatty acids being myristic, palmitic and stearic acids. Polysorbate 80 was super refined and purchased from Croda (Mill Hall, PA, USA). Polysorbate 80 had 87% oleic acid, with the other major fatty acids being palmitic, stearic and linoleic (0.4%). 5,5-Dimethyl-1-pyrroline-N-oxide (DMPO, 97%) was purchased from Combi Blocks (San Diego, CA, USA). Ultrapure water (18.2 MΩ) was used for diluting the samples. Glass capillaries were purchased from Drummond Scientific Company (Broomall, PA, USA). EPR Suprasil tubes (5 mm inner diameter) were purchased from Wilmad LabGlass (Vineland, NJ, USA). Cha-Seal Tube sealing compound was purchased from Kimble (Millville, NJ, USA).

Experiments were conducted on a Bruker EMXnano X-band benchtop EPR spectrometer (Billerica, MA, USA) equipped with a heater transfer line with nitrogen gas flow and a UV light source that was controlled by Xenon version 001 software. A total of 300 µL of the sample was mixed with 3 µL of DMPO, then transferred to a 40 µL capillary when an aqueous solution was used and a 100 µL capillary when a stock polysorbate was used, then sealed. The capillary was placed in a 5 mm I.D. Suprasil tube. The tube was left in the spectrometer for 10 min at 50 °C. A 1D field sweep measurement was performed with the following parameters: sweep width 100 G, center field 3439 G, gain 40 db, modulation amplitude 0.7 G, sweep time 20 s, X-band microwave frequency 9.6503 GHz, microwave power 10 mW, time constant 10.24 ms, number of points 1429, and 25 scans. The obtained spectra were fitted with simulated spectra using the SpinFit module included in Xenon software for stocks and solutions alike. Fitting parameters were optimized to start the fitting of each spectrum with a search for 5 DMPO adducts, each with a starting g factor of 2.007. A second order fitting was applied, as well as fitting of the line position, width and shape. The starting hyperfine coupling constants for SpinFit are shown in Table 2. The concentration of each radical was determined using the SpinCount feature in the Xenon software. The spectral features that match in the simulated spectra and the measured spectra are shown in Figures S2 and S3. The quality of the fit was demonstrated by the clear residual plots shown in Figure S4.

## 5. Conclusions

The suggested mechanism from this study provides an improved understanding of the oxidative degradation pathways of PSs. Since the same radical species were identified in PS20 and PS80, it is suggested that the main oxidation pathway is similar for both PSs and not focused on the carbon–carbon double bond; this double bond likely only increases the rate of oxidation by adding an additional oxidation pathway rather than being the main pathway. To mitigate the oxidative degradation of PS, attention must be given to initial peroxide levels in the PS as well as to the formation of hydroxyl radicals in the storage solution. This is because the initiation of oxidation appears to come from the initiating species already present in the polysorbate raw material. The differences in the radical species identified in the ‘pure’ PS stocks and when in an aqueous solution demonstrate that degradation studies looking at aqueous solutions will have different outcomes than if they were looking at the initial stock. Most analytical methods for studying PS require dissolving the PS, thus altering the radical species present and the degradation pathway. Furthermore, the diluent of the characterization method, in addition to the PS sample handling, will impact the rate and type of degradation observed. Due to the exquisite sensitivity to organic radicals and the ability to study the raw PS material, EPR spectroscopy is ideally suited to studying stock PSs and thus can indicate the stability of the initial PS reagent before it is used in the manufacturing process.

## Figures and Tables

**Table 1:** Hyperfine coupling constants (hfc) of DMPO radical adducts identified in PS stocks (both PS20 and PS80) and 10% aqueous solutions of the stocks. n = 4, except for R^•^ which was n = 10 and PEO-R^•^ which was n = 6. g factors were 2.0074 ± 0.0003.

Radical Species	Hyperfine Coupling Constant (G)			
	*a*_N_	*a*_H__β_	*a*_H__γ_	References
PS stocks				
R-OO^•^	13.8 ± 0.1	11.2 ± 0.2	0.7 ± 0.2	[30,31]
R-O^•^	13.4 ± 0.1	8.2 ± 0.8	1.4 ± 0.1	[30,31,32,33,35]
R^•^	14.7 ± 0.2	19.8 ± 0.3	-	[33,35]
DMPO-X	14.3 ± 0.5	-	-	[28,31]
10% aqueous PS solutions				
HO^•^	15.0 ± 0.1	14.3 ± 0.2	-	[28]
R-O^•^	12.6 ± 0.1	9.5 ± 0.3	3.9 ± 1.5	[30,31,32,33,35]
PEO-R^•^	15.8 ± 0.1	22.3 ± 0.3	-	[28,36,37]
DMPO-X	14.6 ± 0.1	-	-	[28,31]

**Table 2:** Starting fitting hyperfine coupling constants for polysorbate-DMPO EPR spectra.

Adduct Species	Hyperfine Coupling Constant (G)		
	*a*_N_	*a*_H__β_	*a*_H__γ_
1	14.3	20.2	
2	13.9		
3	14.7	19.5	
4	13.2	7.7	1.5
5	13.7	11	1.0

## Data Availability

The data presented in this study are available on request from the corresponding author.

## Supplementary Materials

The following supporting information can be downloaded at: https://www.mdpi.com/article/10.3390/ph17020233/s1, Figure S1 EPR spectra of 10% aqueous PS80 with DMPO, as well as simulations of spectra from DMPO radical adducts of •OH, R•, R-O• and DMPO-X. Figure S2 EPR spectra and simulations of stock PS20 and PS80 with DMPO shown in Figure 2 with lines to indicate spectral features. Figure S3 EPR spectra and simulations of 10% aqueous PS20 and PS80 with DMPO shown in Figure 3 with lines to indicate spectral features. Figure S4 residual plots after simulating the EPR spectra for both the stocks and polysorbate solutions.
