# Supplementary material for: Determining the Oxidation Mechanism through Radical Intermediates in Polysorbates 80 and 20 by Electron Paramagnetic Resonance Spectroscopy
Source: Pharmaceuticals (Basel). 2024 Feb 9;17(2):233. doi: 10.3390/ph17020233 (PMC10892813; doi:10.3390/ph17020233)
Supplement: Supplementary file 1 [file pharmaceuticals-17-00233-s001.zip › pharmaceuticals-2810586-supplementary.pdf]

Supplementary Information

Determining the Oxidation Mechanism through Radical Intermediates in Polysorbates 80 and 20 by Electron Paramagnetic Resonance Spectroscopy

Adam T. Sutton and Richard R. Rustandi

Analytical Research & Development, Merck & Co., Inc., Rahway, NJ 07065, United States

Correspondence: adam.sutton@merck.com

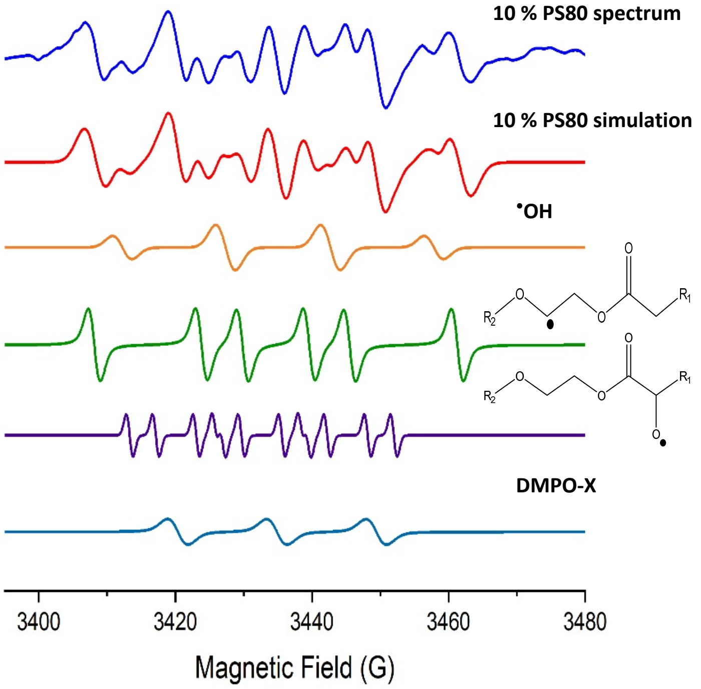

Figure S1. EPR spectra of 10% aqueous PS80 with DMPO, as well as simulations of spectra from DMPO radical adducts of •OH, R•, R-O• and DMPO-X.

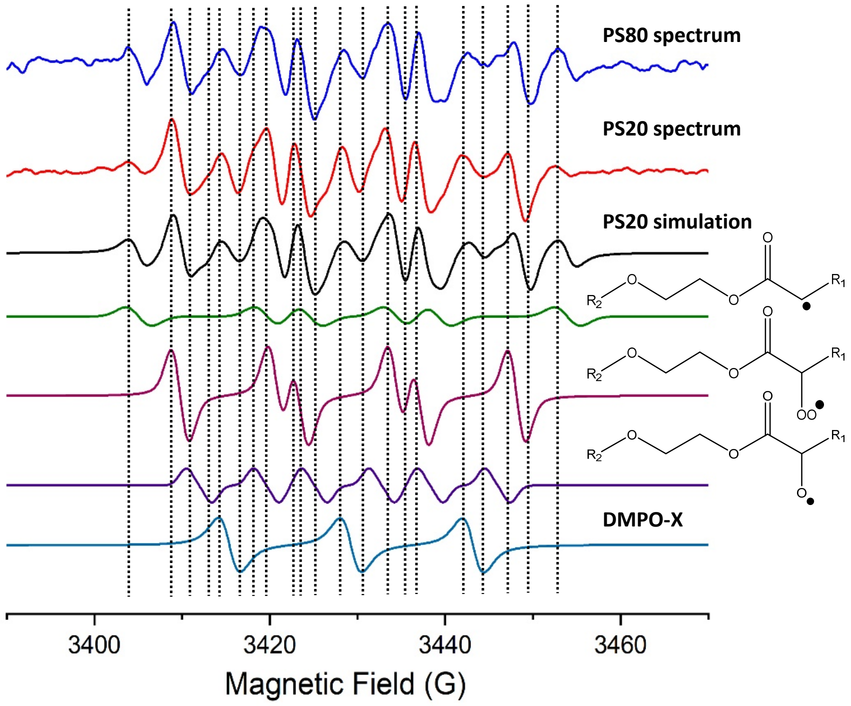

Figure S2. EPR spectra and simulations of stock PS20 and PS80 with DMPO shown in Figure 2 with lines to indicate spectral features.

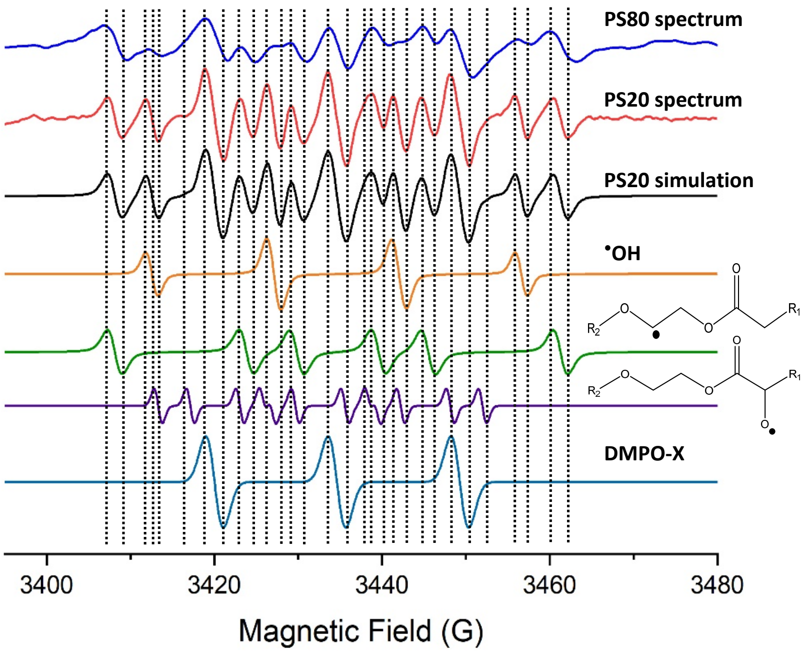

Figure S3. EPR spectra and simulations of 10% aqueous PS20 and PS80 with DMPO shown in Figure 3 with lines to indicate spectral features.

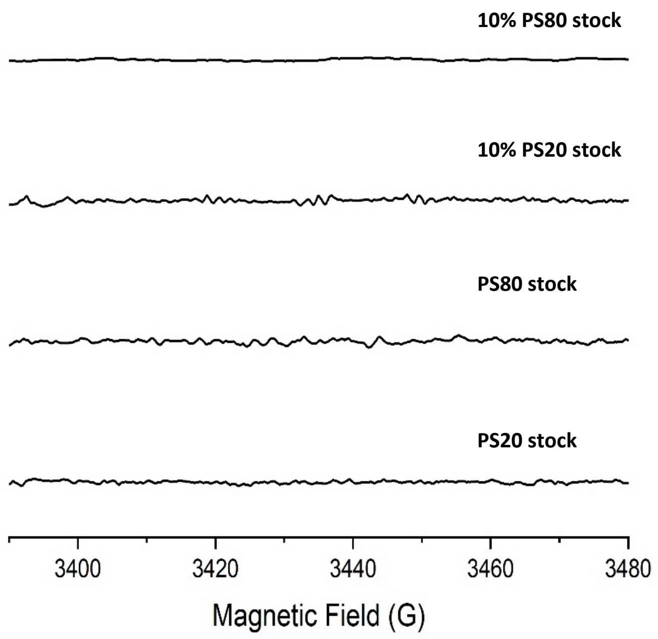

Figure S4. Residual plots after simulating the EPR spectra for both the stocks and polysorbate solutions.
